# Dr. John Hougham Bell

**Published:** 1910-07-16

**Authors:** 


We have to record the death of Dr. John Hougham Bell at the age of sixty-four. He studied at King's College, Edinburgh, and Paris, and took his M.R.C.S. in 1866, graduating M.D. of Edinburgh in 1867. His appointments included the post of assistant medical superintendent to St. Andrew's Hospital for Mental Diseases, Northampton, and resident clinical assistant to the Bethlehem Royal Hospital. He was also surgeon to the Royal National Consumption Hospital, Ventnor.

